# Sex-specific variations in global DNA methylation levels with age: a population-based exploratory study from North India

**DOI:** 10.3389/fgene.2023.1038529

**Published:** 2023-05-15

**Authors:** Anshika Kaushik, Vineet Chaudhary, Imnameren Longkumer, Kallur Nava Saraswathy, Sonal Jain

**Affiliations:** ^1^ Laboratory of Molecular and Biochemical Anthropology, Department of Anthropology, University of Delhi, Delhi, India; ^2^ Department of Anthropology, University of Delhi, Delhi, India

**Keywords:** global DNA methylation, aging, epigenetics, epigenetic drift, epigenetic clock

## Abstract

**Purpose:** Aging is one of the most important risk factors for a number of human diseases. Epigenetic alterations, including changes in DNA methylation patterns, have been reported to be one of the hallmarks of aging. Being a malleable process, the role of site-specific DNA methylation in aging is being extensively investigated; however, much less attention has been given to alterations in global DNA methylation with aging at the population level. The present study aims to explore overall and sex-specific variations in global DNA methylation patterns with age.

**Methods:** A total of 1,127 adult individuals (792 females) aged 30–75 years belonging to Haryana, North India, were recruited. Socio-demographic data was collected using a pretested interview schedule. Global DNA methylation analysis, of peripheral blood leucocyte (PBL) DNA, was performed using the ELISA-based colorimetric technique.

**Results:** Though the overall correlation analysis revealed a weak inverse trend between global DNA methylation and age, the adjusted regression model showed no significant association between global DNA methylation and age. In age-stratified analysis, global DNA methylation levels were found to be fairly stable until 60 years of age, followed by a decline in the above-60 age group. Further, no significant difference in DNA patterns methylation pattern was observed between males and females.

**Conclusion:** Overall, the study suggests a lack of association between global DNA methylation and age, especially until 60 years of age, and a similar DNA methylation pattern between males and females with respect to age.

## 1 Introduction

Aging is a multifaceted and time-dependent deterioration of the physiological processes encountered by most living organisms (López-Otín et al., 2013). The process of aging and associated underlying biological mechanisms have intrigued the human mind since the beginning; however, advancement in technology has now enabled us to understand them at the molecular level (López-Otín et al., 2013; Pal & Tyler, 2016). Aging is the most important risk factor for several human diseases like cancers, cardiovascular diseases (CVDs), and neurodegenerative disorders among others (Pagiatakis et al., 2021). Owing to significant development in medical and public health resources, human life expectancy has augmented rapidly in the last few decades; however, enhanced life expectancy has resulted in higher morbidity and years lived in disability (Pagiatakis et al., 2021). Therefore, it is pertinent to understand the aging process so that adverse health outcomes associated with it can be minimized.

Studies have identified certain hallmarks of aging, viz genomic instability, telomere shorting, loss of proteostasis, etc., epigenetic alterations being one among those hallmarks (López-Otín et al., 2013). Being reversible, at least theoretically, epigenetic alterations associated with aging are being extensively studied to explore the possibilities of healthy aging (Jones et al., 2015). DNA methylation, by far, is the most widely studied epigenetic process (Pal & Tyler, 2016). DNA methylation refers to the addition of a methyl group at the fifth carbon of the cytosine residues (5 mC) of CpG dinucleotides (cytosine proximal to guanine) (Martin & Fry, 2018). Generally, DNA methylation occurs within those genomic regions with high cytosine and guanine (CG) content, the so-called CpG islands (Martin & Fry, 2018); however, CpH (H = A, T, or C) sites can also be methylated (Lister et al., 2013). DNA methylation patterns are established by DNA methyltransferases (DNMTs), mainly DNMT3a, DNMT3b, and DNMT1 (Unnikrishnan et al., 2018). While DNMT3a and DNMT3b are *de novo* methyltransferases that can recognize and methylate both hemimethylated and unmethylated DNA, DNMT1 is a maintenance methyltransferase capable of functioning only on hemimethylated DNA (Okano et al., 1999; Unnikrishnan et al., 2018).

DNA methylation levels can be affected by both intrinsic (viz genetic background) and extrinsic factors (like smoking, diet, exposure to air pollution, certain chemicals, etc.) (Gopalan et al., 2017; Martin & Fry, 2018). Besides these factors, aging has been reported to influence DNA methylation levels (Gopalan et al., 2017). Aging and longevity have directly been linked with DNA methylation and epigenetic alterations in humans and several other organisms, the general trend being increased global hypomethylation and regions of hypermethylation with age (Johnson et al., 2012). According to the genomic hypomethylation hypothesis, global DNA methylation decreases with age, resulting in decreased genomic stability and abnormal gene expression (Unnikrishnan et al., 2018). Though the theory of genomic hypomethylation with age is still popular, recent studies employing modern quantitative techniques have challenged it (Lister et al., 2013; Unnikrishnan et al., 2018).

In one of the earliest attempts to explore the relationship between global DNA methylation and aging, Vanyushin et al. (1973) studied the changes in 5 mC content of DNA extracted from different tissues of male albino rats over the age range between 1 and 28 months. They reported a decrease in the 5 mC content of DNA extracted from brain, heart, and spleen tissues with age; however, no change in the 5 mC content of DNA extracted from liver, lung, and kidney tissues (Vanyushin et al., 1973). In a landmark paper, Wilson and Jones (1983) reported a decrease in 5 mC content with increased population doublings (replicative senescence) in DNA extracted from skin fibroblasts of mice, hamsters, and humans. In another study, Wilson et al. (1987) reported an age-related decline in the 5 mC content of DNA extracted from the brain, liver, and small intestine of mice, the liver and small intestine of *Peromyscus,* and the bronchial epithelial cells of humans. Studies so far used thin-layer chromatography (TLC) for the estimation of 5 mC content. Later, Fuke et al. (2004) used High-pressure liquid chromatography (HPLC) to study the age-related change in 5 mC content in peripheral leukocytes and placentas of humans. They reported an age-related decrease in the 5 mC content of DNA extracted from peripheral leukocytes but a gestational stage-dependent increase in the 5 mC content of placentas (Fuke et al., 2004).

Several studies have also used antibody-based Enzyme-Linked Immunosorbent Assays (ELISA) technique to study the relationship between DNA methylation and aging in animal models, with some studies reporting an age-dependent decrease in methylation (Mei et al., 2015; Tong et al., 2015), while others reporting either an increase or no change in DNA methylation with age (Chen et al., 2012; Chouliaras et al., 2012; Armstrong et al., 2014). More recent studies have used next-generation sequencing (NGS) to study the effects of age on the 5 mC content among humans and animals and have almost unanimously rejected the age-related genomic hypomethylation hypothesis in favour of no association between global 5 mC content and age (Lister et al., 2013; Raddatz et al., 2013; Sun et al., 2014; Hadad et al., 2016; Cole et al., 2017)

However, despite the growth in the number of studies investigating the relationship between age and DNA methylation, there is a paucity of population-specific studies exploring age-related alterations in global DNA methylation patterns. Being cheaper than gene-specific methylation analysis, global DNA methylation analysis can be an economical alternative to understanding age-related epigenetic variations and trends at population levels, as well as developing epigenetic standards of healthy aging. The present population-based study aims to understand the variations in global DNA methylation levels with age and the role of sex in the modulation of the relationship between them.

## 2 Materials and methods

### 2.1 Study area and participants

The data for the present cross-sectional study has been derived from a major research project funded by the Department of Biotechnology, Government of India. A total of 1,127 adult individuals of either sex (70.3% females) aged 30 to 75 and belonging to an endogamous community (Jat community) were recruited from 15 villages of Palwal district, Haryana, North India. Jat is a large ethnic community of North and Northwest India having a sizable population in Haryana, Delhi, Punjab, Western Uttar Pradesh, Rajasthan, and Gujarat. Jat community practices community endogamy (i.e., they marry within the Jat community) but village exogamy (males marry outside their villages).

Individuals with any chronic or terminal disease (CVDs, cancers, diabetes, etc.), major infectious, severe mental disorders, lactating mothers, and pregnant women were excluded from the study as these conditions can influence the DNA methylation patterns. Further, up to second-degree relatives of the recruited participants were also excluded.

Informed written consent was obtained from every participant in their local language prior to their recruitment. The study protocol was evaluated and approved by the institutional ethics committee, Department of Anthropology, University of Delhi (Ref No. Anth/2010/455/1).

### 2.2 Data collection

Socio-demographic and lifestyle data (age, sex, literacy, alcohol intake, smoking, and diet) were collected using a pre-tested and modified interview schedule. A sample of 5 mL overnight fasting venous blood was collected from each participant by a trained technician. DNA extraction was done using the salting-out method (Miller et al., 1988). DNA quality check was performed using a nanodrop spectrophotometer with A260/A280 ∼ 1.8 (1.6–2.0) as a standard. DNA samples were stored at −80°C till further analysis.

### 2.3 Global DNA methylation analysis

Previously extracted peripheral blood leucocyte (PBL) DNA was used for global DNA methylation analysis. Global DNA methylation levels were estimated using Epigentek Methylflash™ kits (Epigentek Group Inc., New York, United States) working on the ELISA-based colorimetric technique. Antibodies provided with the kit were used to detect the 5-methylcytosine content of the DNA sample, followed by quantification at 450 nm using a spectrophotometer (MultiscanGo Spectrophotometer, Thermo Fisher Scientific, Waltham, Massachusetts, United States). Methylation assays were performed in duplicates and for each assay 200 ng of DNA samples were used. Variations in Intra- and inter-assay coefficients were found to be less than 5%. The global DNA methylation assay was performed by a single individual to avoid handling variation.

### 2.4 Statistical analysis

SPSS version 22.0 (IBM-SPSS Inc. Chicago, IL) was used for the statistical analysis. Continuous variables were subjected to the Kolmogorov-Smirnov normality test, and median levels with IQR have been reported for non-normally distributed variables. Mann-Whitney U and Kruskal–Wallis H tests were used for comparing median levels of two and more than two groups, respectively. The *t*-test was used to compare the mean levels of the two groups. Further, the participants were grouped into 10-year age cohorts, and the correlation of global DNA methylation level with age in the entire sample and within each cohort was calculated using the Spearman’s correlation test. Adjusted linear regression was used to study the variation in methylation levels with age in the entire study sample as well as within the age cohorts. A *p*-value < 0.05 was regarded as statistically significant. Further, since some of the analyses involved multiple testing, the *p*-value threshold at *α* = 0.05, after correcting for four tests, was taken as *p* = 0.0125.

## 3 Results

### 3.1 General characteristics of the study participants

The distribution of sociodemographic and lifestyle variables among the participants is presented in Table 1. The proportion of males in older age categories, non-vegetarian category, among literates, smokers, and drinkers was found to be significantly higher than that of females; therefore, these variables were adjusted while performing the regression analysis.

**TABLE 1:** Distribution of sociodemographic and lifestyle variables among the participants.

Variables		Overall (N = 1,127)	Males (N = 335)	Females (N = 792)	*p*-value^α^
		n (%)	n (%)	n (%)	
Mean age ± SD (in years)		48 ± 10	51.3 ± 9.9	46.6 ± 9.8	<0.001*^,ζ^
Age cohorts (in year)	30–40	243 (21.6)	35 (10.4)	208 (26.3)	<0.001*
	40–50	398 (35.3)	113 (33.7)	285 (36)	
	50–60	275 (24.4)	100 (29.9)	175 (22.1)	
	≥60	211 (18.7)	87 (26)	124 (15.7)	
Diet	Veg	1,047 (93)	278 (83)	769 (97.2)	<0.001*
	Non-veg	79 (7)	57 (17)	22 (2.8)	
Literacy	Illiterate	687 (61)	84 (25.1)	604 (76.3s)	<0.001*
	Literate	440 (39)	251 (74.9)	188 (23.7)	
Smoking	Smokers	623 (55.3)	272 (81.2)	351 (44.3)	<0.001*
	Non-smokers	504 (44.7)	63 (18.8)	441 (55.7)	
Alcohol	Drinkers	93 (8.2)	93 (27.8)	0 (0)	<0.001*
	Non-drinkers	1,034 (91.8)	242 (72.2)	792 (100)	

^*^Significant at *p*-value <0.05.

^α^male vs. females.

^ζ^t-test *p*-value.

### 3.2 Variation in global DNA methylation with age

To understand the variations in global DNA methylation levels with age, participants were divided into 10-year age cohorts, and the median global DNA methylation level was calculated for each cohort (Table 2). Median global DNA methylation levels of the first three age cohorts were found to be comparable, indicating a phase of stability with respect to DNA methylation levels between 30 and 60 years of age. The median global DNA methylation level of the next age cohort (≥60 years) was found to be relatively lower than those of the previous age cohorts.

**TABLE 2:** Variation in global DNA methylation with age.

Age cohorts (in year)	Median global DNA methylation (IQR)	*p*-value	Correlation coefficient (ρ)	*p*-value	β (SE)^α^	*p*-value
Whole sample (N = 1,127)	0.63 (0.33–1.22)	—	−0.065	0.03*	−0.004 (0.005)	0.34
30–40 (N = 243)	0.67 (0.38–1.21)	0.013*	0.048	0.457	0.027 (0.042)	0.53
40–50 (N = 398)	0.65 (0.36–1.28)		−0.012	0.811	−0.006 (0.025)	0.80
50–60 (N = 275)	0.60 (0.33–1.3)		0.179	0.003*	0.068 (0.038)	0.08
≥60 (N = 211)	0.54 (0.22–1.04)		−0.139	0.043*	−0.054 (0.025)	0.03*

^*^Significant at *p*-value <0.05.

^α^adjusted for sex, smoking, alcohol, diet, and literacy.

Further, the correlation and association of global DNA methylation with age in the whole study sample and also within different age cohorts were studied using Spearman’s correlation and linear regression, respectively (Table 2). Though Spearman’s correlation analysis showed a weak but statistically significant inverse correlation between global DNA methylation and age, the adjusted regression model found no significant association between them in the overall analysis. Again, in age cohort-wise analysis, no statistically significant relationship was observed between global DNA methylation and age, except in the age cohort ≥60 years, where the negative association between global DNA methylation level and age was statistically significant (at *p*-value threshold of 0.05 but not at *p*-value threshold of 0.0125).

### 3.3 Gender-specific variation in global DNA methylation with age

**FIGURE 1:**
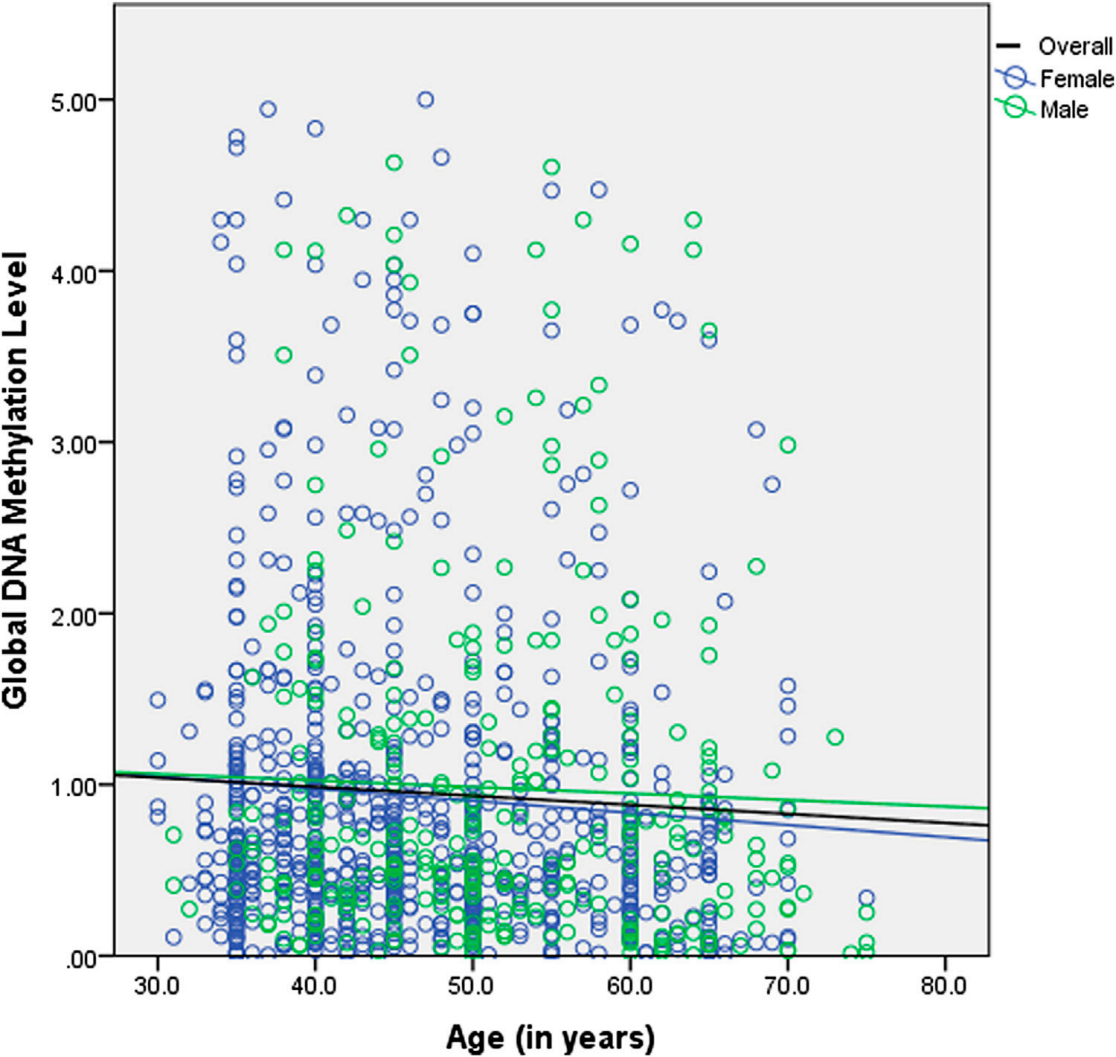
Scatter-plot between global DNA methylation and age.

The effect of gender on variations in median global DNA methylation with age was assessed in the whole study sample and also within different age cohorts (Table 3) (Figure 1). No statistically significant difference was found between the median global DNA methylation levels of males and females in both overall as well as the age cohort-wise analyses.

**TABLE 3:** Gender-wise variation in global DNA methylation with age.

Age cohorts (in years)	Gender	Median global DNA methylation (IQR)	*p*-value^a^	Correlation coefficient (ρ)	*p*-value	β (SE)^ζ^	*p*-value
Whole sample	Male (N = 335)	0.64 (0.3–1.37)	0.584	−0.065	0.238	−0.010 (0.009)	0.210
	Female (N = 792)	0.62 (0.35–1.18)		−0.076	0.033*	−0.002 (0.005)	0.718
30–40	Male (N = 35)	0.61 (0.32–1.19)	0.417	0.111	0.524	0.069 (0.078)	0.379
	Female (N = 208)	0.69 (0.38–1.23)		0.047	0.498	0.019 (0.048)	0.688
40–50	Male (N = 113)	0.66 (0.39–1.45)	0.318	−0.048	0.610	−0.065 (0.056)	0.248
	Female (N = 285)	0.64 (0.36–1.19)		−0.004	0.949	0.018 (0.027)	0.504
50–60	Male (N = 100)	0.75 (0.37–1.77)	0.165	0.354	<0.001*	0.187 (0.067)	0.007*
	Female (N = 175)	0.59 (0.31–1.2)		0.080	0.292	0.016 (0.045)	0.720
≥60	Male (N = 87)	0.56 (0.13–1.1)	0.835	−0.151	0.162	−0.049 (0.032)	0.137
	Female (N = 124)	0.52 (0.25–0.97)		−0.129	0.155	−0.056 (0.036)	0.120

^*^Significant at *p*-value <0.05.

^a^males vs. females.

^ζ^adjusted for smoking, alcohol, diet, and literacy.

Further, the whole study sample, as well as each age cohort, was stratified for gender to understand the gender-specific correlation and association of global DNA methylation with age (Table 3). The correlation analysis between global DNA methylation and age yielded a weak negative but significant coefficient among females in the overall analysis. However, in the overall as well as the age cohort-wise analyses, the adjusted regression model showed no significant association between global DNA methylation and age in either sex, except for males in the age group 50–60 years. Global DNA methylation was found to be positively associated with age among males in 50–60 age group.

## 4 Discussion

Of various hallmarks of aging, epigenetic alterations have been of particular interest to the scientific community due to their reversible nature (López-Otín et al., 2013). A monumental growth in both the quantity and quality of studies exploring the epigenetics of aging has been witnessed in the last 2 decades (Pal & Tyler, 2016; Unnikrishnan et al., 2019; Pagiatakis et al., 2021). However, much less attention has been given to age-related alterations in global DNA methylation levels in healthy populations (Tsang et al., 2016). The present population-based study has attempted to understand the trends of PBL global DNA methylation levels with age and the role of gender in modulating this relationship.

In the present study, the median global DNA methylation remained fairly stable between 30 and 60 years of age, followed by a relative decline in the above-60 years age cohort. The adjusted regression model revealed a lack of statistically significant association between global DNA methylation and age in the overall as well as age cohort-stratified analysis, except in the above-60 years age cohort. In the above-60 years age cohort, DNA methylation was found to be negatively associated with age (at *p*-value threshold of 0.05 but not at *p*-value threshold of 0.0125). Though these findings are partly in concordance with the trend of no age-related changes in global DNA methylation levels reported in some of the recent NGS-based studies (Lister et al., 2013; Raddatz et al., 2013), other studies (employing TLC and HPLC) have reported a general decrease in 5 mC content (Wilson et al., 1987; Fuke et al., 2004). Further, the trend of decreased 5 mC content with age among older participants in the present study is consistent with some of the previous studies (Bollati et al., 2009; Tsang et al., 2016). The trend of decline in global DNA methylation with age among older adults is the most consistent one (Bollati et al., 2009; Zampieri et al., 2015; Pal & Tyler, 2016).

In recent times, the general consensus viz-a-viz age-related changes in global DNA methylation has shifted from genomic hypomethylation to no change (Masser et al., 2018). However, conflicting findings have also been reported (Bollati et al., 2009; Tsang et al., 2016). One of the reasons behind conflicting reports can be differences in the technology used to estimate the 5 mC content, and the other reason could be the difference in the tissue used for DNA isolation (Unnikrishnan et al., 2018).

Again, though several NGS-based studies (Lister et al., 2013; Raddatz et al., 2013; Sun et al., 2014; Hadad et al., 2016; Cole et al., 2017), as well as the present study (ELISA-based), have not found any significant association between global DNA methylation levels and aging, the importance of epigenetics in the aging process cannot be undermined (Unnikrishnan et al., 2019; Salameh et al., 2020). Studies have reported reduced DNA methylation levels and increased variability in repetitive sequences like Alu and HERV-K with age (Bollati et al., 2009; Jintaridth & Mutirangura, 2010; Zampieri et al., 2015). However, not all repeated sequences have been reported to be affected equally by age-dependent hypomethylation (Zampieri et al., 2015). Hypomethylation has been reported to occur in Alu and HERV-K at different ages; however, LINE-1 sequences are not affected significantly (Zampieri et al., 2015). Studies exploring age-related changes in genes specific methylation patterns have shown progressive hypermethylation, mostly in the promoter region marking the loss of gene expression (Zampieri et al., 2015). Such gene-specific hypermethylation have been associated with a number of diseases like cancers, cardiovascular diseases, Alzheimer’s disease, etc. (Zampieri et al., 2015; Pagiatakis et al., 2021).

While age-related epigenetic alterations are being studied extensively, the underlying reasons behind these alterations remain unanswered (Jones et al., 2015). It is believed that both environmental and stochastic factors contribute to age-related epigenetic changes, though their relative contributions to epigenetic drift (variability in epigenetic patterns with age) and epigenetic clock (consistent age-related epigenetic changes) are unknown (Jones et al., 2015). According to the mitotic clock hypothesis, every successive division of a stem cell is associated with the possibility of errors in the maintenance of the DNA methylation state by methyltransferase enzymes (Issa, 2014). Due to its random and probability-driven nature, the mitotic clock hypothesis can well explain age-related stochastic methylation drift but fails to explain predictable and conserved age-related epigenetic clock (Zheng et al., 2016). In fact, existing hypotheses do not sufficiently explain the direction of changes in DNA methylation levels (Unnikrishnan et al., 2019).

Besides age, a number of sociodemographic and lifestyle variables, like diet, smoking, alcohol consumption, and education/literacy, have also been reported to influence DNA methylation levels (Lim & Song, 2012; van Dongen et al., 2018). An elaborate discussion on the relationship between these variables and DNA methylation levels is out of the scope of the present paper; nevertheless, it is worth mentioning that the effect of these variables on DNA methylation levels was statistically adjusted while computing the association between age and PBL DNA methylation levels.

Coming to the role of sex in age-related changes in 5 mC content, sex appeared to play a limited role in modulating the relationship between global DNA methylation and age in the present study. The difference in median global DNA methylation levels between males and females was not found to be statistically significant in overall as well as age-stratified analyses. Only a few studies, thus far, have explored the effect of sex on alterations in DNA methylation with age (Unnikrishnan et al., 2019). In general, sex is not believed to influence age-related epigenetic changes at the whole genome level, especially in below 60 age groups (Yusipov et al., 2020). However, specific genomic sites do show sex-specific age-related epigenetic alterations (Yusipov et al., 2020). In a previous study, sex was reported to influence age-related DNA methylation changes only among older age groups (Tsang et al., 2016). One observation in the present study that requires cautious interpretation is the positive association between DNA methylation and age among males in the 50–60 age group. Though Tsang et al. (2016) have reported a somewhat similar finding, the reason behind the positive association between DNA methylation and age among males of this age group is not apparent.

The present study has certain limitations that must be stated. Firstly, the proportion of females is twice that of males, which may have affected the overall results. However, to neutralize the confounding effect of sex, all the analyses that have been performed on the overall sample have also been performed on females and males separately. Further, being a single-sited study, the generalization of the results may be difficult.

## 5 Conclusion

In the present study, though a nominal inverse trend was observed between global DNA methylation and age, the association between them was not found to be statistically significant in the adjusted model. When stratified for age, global DNA methylation level was found to be fairly stable until the age of 60, followed by a relative decline in the above-60 age group. Also, DNA methylation was found to be negatively associated with age in the above-60 years age cohort (though the observed relationship could not reach the level of statistical significance after Bonferroni correction). Further, no statistically significant difference between the median global DNA methylation levels of males and females was observed in the whole sample or any of the age cohorts. More studies should be taken up to further explicate the variations in DNA methylation levels with age and also to understand the role of sex in modulating the relationship between them.

## Data Availability

The raw data supporting the conclusion of this article will be made available by the authors, without undue reservation.
